# The *DFR* locus: A smart landing pad for targeted transgene insertion in tomato

**DOI:** 10.1371/journal.pone.0208395

**Published:** 2018-12-06

**Authors:** Benoit Danilo, Laura Perrot, Emmanuel Botton, Fabien Nogué, Marianne Mazier

**Affiliations:** 1 INRA PACA, UR 1052, GAFL unit (Génétique et Amelioration des Fruits et Légumes), Avignon, France; 2 Institut Jean-Pierre Bourgin, INRA, AgroParisTech, CNRS, Université Paris-Saclay, Versailles, France; Ecole Normale Superieure, FRANCE

## Abstract

Targeted insertion of transgenes in plants is still challenging and requires further technical innovation. In the present study, we chose the tomato *DFR* gene involved in anthocyanin biosynthesis as a landing pad for targeted transgene insertion using CRISPR-Cas9 in a two-step strategy. First, a 1013 bp was deleted in the endogenous *DFR* gene. Hypocotyls and callus of *in vitro* regenerated plantlets homozygous for the deletion were green instead of the usual anthocyanin produced purple colour. Next, standard *Agrobacterium*-mediated transformation was used to target transgene insertion at the *DFR* landing pad in the *dfr* deletion line. The single binary vector carried two sgRNAs, a donor template containing two homology arms of 400 bp, the previously deleted *DFR* sequence, and a *NptII* expression cassette. Regenerating plantlets were screened for a purple-colour phenotype indicating that DFR function had been restored. Targeted insertions were identified in 1.29% of the transformed explants. Thus, we established an efficient method for selecting HDR-mediated transgene insertion using the CRISPR-Cas9 system in tomato. The visual screen used here facilitates selection of these rare gene targeting events, does not necessitate the systematic PCR screening of all the regenerating material and can be potentially applied to other crops.

## Introduction

CRISPR-Cas9 (Clustered Regularly Interspaced Short Palindromic Repeats/CRISPR-associated protein) technology surpasses other genome editing tools such as zinc finger nucleases (ZFNs), meganucleases and TAL effector nucleases (TALENs) due to numerous advantages in terms of cost, ease of use and efficiency of targeting DNA sequences [1].

The CRISPR-Cas9 system is based on RNA-protein interactions involving a single non-coding guide RNA (sgRNA) and the Cas9 nuclease derived from *Streptococcus pyogenes*, *aureus* or *thermophilus*. Double-stranded breaks (DSBs) induced by the CRISPR-Cas9 system are repaired using either of two mechanisms: i) non-homologous end joining (either the classical non-homologous end-joining reaction C-NHEJ or an alternative end-joining reaction alt-EJ, also called microhomology-mediated end joining, MMEJ), that can be error-prone and introduce mutations including deletions or ii) homology-directed repair (HDR) that needs a repair template with homology to the targeted sequence and can lead to precise gene knock-in or insertion. [2]. The application of the CRISPR-Cas9 system has increased exponentially since 2013. The majority of plant studies report an efficient use of the error-prone non-homologous end-joining mechanism to introduce short deletions or random insertions (indels) at the targeted site in order to generate a gene knock-out. In tomato, several reports validated the CRISPR-Cas9 system as an effective tool to knock-out genes involved in various functions such as leaf development [3], rhizogenesis [4], drought tolerance [5], meristem size [6], pollen development [7], tomato inflorescence architecture [8–10], fruit characteristics such as ripening [11–14], parthenocarpy [15,16], size [17], content [18,19] or colour [20], as well as sensitivity to pathogens [21].

Although gene knock-out can be used for trait improvement, the most appealing approach for plant engineering and breeders is gene knock-in for precise modifications, base or gene replacement, targeted integration of a gene of interest or pyramiding of several genes in elite genotypes without the undesirable effects associated with random transgene insertion in the genome. The first demonstrations of successful HDR-mediated gene knock-in using CRISPR-Cas9 were achieved in tobacco and rice protoplasts by targeting the *PDS* gene [22,23]. In 2014, Schiml et al. succeeded in generating stable insertion of a DNA donor template in the *ADH1* locus in *Arabidopsis* and confirmed that CRISPR-Cas9 nuclease and nickase systems can be used for HDR-mediated gene targeting [24]. HDR-mediated gene targeting were obtained *via Agrobacterium* transformation in cassava and rice with an efficiency close to 1% [25,26]. In tomato an HDR-mediated modification of a single nucleotide in the *ALC* gene involved in the long-shelf-life properties was reported for two plants [27]. Other HDR-mediated gene targeting experiments were reported using particle bombardment delivery [28].

HDR-mediated gene targeting remains challenging because NHEJ is the favoured mechanism for DSB DNA repair in plants and higher organisms [29]. Improvements focused on the DNA donor template and alternative ways of delivery were investigated, see review [30,31]. Recently in tomato, a deletion of 281 bp was repaired through HDR in 25% of the plants using a repair DNA template amplified *via* a geminiviral replicon [32]. It is the highest efficiency of HDR obtained until now.

Besides optimization to increase HDR frequency, which rarely exceeds a few percent, another approach would be to improve the detection of these very rare HDR events. The use of selection markers or reporter genes could be helpful for further protocol optimization. Plant markers for HR-directed repair without any effect on plant growth and development were reported in *Arabidopsis* [33]. In tomato, the replacement of the promoter upstream of the *ANT 1* gene, which encodes a MYB factor involved in anthocyanin biosynthesis, with the cauliflower mosaic virus 35S promoter, resulted in a constitutive strong purple colour easy to screen visually but unfortunately not usable for plant breeding [34].

Few attempts to exploit the anthocyanin pathway for genome editing purposes were mentioned in the literature [34–37]. The knock-out of the *F3H* gene in carrot resulted in an absence of purple colouration in the *in vitro* regeneration events providing a visual marker for screening the mutated events [37]. In Japanese morning glory *Ipomoea (Pharbitis) nil*, knock-out of the *DFR-B* gene led to changes in stem colour during the early stages of plant tissue culture and also resulted in variations in flower colour [36]. The gene encoding for the DFR protein (dihydroflavonol 4-reductase) is involved in anthocyanin biosynthesis and is responsible for the purple pigmentation of hypocotyls in young tomato seedlings [38,39]. *DFR* is a single copy gene localized on chromosome 2 in the tomato genome [38]. The anthocyanin pathway has been well characterised and the *DFR* gene is involved in the reduction of dihydroflavonols to leucoanthocyanidins during the production of the brick-red pelargonidin, red cyanidin and blue delphinidin pigments [40]. Mutations in the *DFR* gene result in a complete absence of anthocyanin pigmentation at all plant stages [38,39]. The fact that complete abolition of DFR activity has no negative impact on plant growth and fertility [36,38,39] makes the *DFR* gene a suitable target for CRISPR-Cas9 modifications and a potential landing pad for transgene insertion into the tomato genome.

In the present study, we report the precise deletion of 1013 bp in the *DFR* gene in WVA106, the genotype in which we plan to perform HR-mediated gene insertion. Homozygous lines carrying this mutation displayed the expected no-anthocyanin phenotype and were used for transgene targeted insertions at the *DFR* landing pad locus. In our strategy, the precise insertion of transgenes by HDR-DNA repair at the landing pad will lead to the recovery of a functional *DFR* gene and thus to restoration of anthocyanin biosynthesis that should be visible as a purple-coloured phenotype as soon as the plants are regenerating *in vitro*. Targeted insertion of a transgene in the tomato *DFR* landing pad was obtained for six independent explants after an *Agrobacterium* mediated-stable transformation with a single binary plasmid carrying both the CRISPR-Cas9 system and a DNA donor template. Our system, or proposed derivatives, provides a straightforward alternative for targeted insertion of transgenes in tomato and can be used to detect and improve HDR-mediated gene engineering.

## Materials and methods

### CRISPR-Cas9 vector construction for deletion and HDR-mediated targeted insertion

The CRISPR-Cas9 vectors used in this study were previously described [41]. The *CAS9* gene present in the pDe-Cas9 vector (kind gift from Holger Puchta, Karlsruhe Institute of Technology) is driven by the constitutive Ubi4-2 promoter from parsley [24]. The pDe-Cas9 plasmid was modified by exchanging the BASTA resistance cassette (1224 bp *Hind*III fragment) with a kanamycin resistance *NptII* cassette (1397 bp *Hind*III fragment amplified by PCR) from pK7WG2D [42] to produce pDe-Cas9-*NptII* or with a hygromycin resistance *Hpt* cassette (1787 bp *Hind*III fragment amplified by PCR) from pH2GW7 [42] to give pDe-Cas9-*Hpt*. The pDe-Cas9-*Hpt* vector was modified by introducing a second Gateway cassette, attR3-ccdB-attR4 in *Eco53kI* to generate the pDe-Cas9-*Hpt*-GT plasmid. Tomato genomic sequences for the U6 and U3 promoters were identified with the Basic Local Alignment Search Tool (respectively coordinates 92150950–92151262 chromosome 1 and 44489659–44489973 chromosome 6) using the *Arabidopsis* U6-26 snRNA (X52528) and U3B snRNA sequences (X52629) as queries [43,44].

Deletion in the *DFR* gene was performed using two sgRNAs. Guide RNAs targeting the tomato *DFR* gene (#Solyc02g085020) were chosen using the CRISPOR website (http://tefor.net/crispor/crispor.cgi) [45]. Two target loci were selected, one in exon 3 (sgRNA-DFR#1, expression driven by the U3 promoter) and one in exon 6 (sgRNA-DFR#2, expression driven by the U6 promoter) of the *DFR* gene. Constructs were designed to create a deletion of 1013 bp in the *DFR* gene. Two target loci were selected for gene insertion, one in the junction of the deletion obtained (sgRNA-DFR#3, expression driven by the U3 promoter) and one at the end of the exon 6 before the STOP codon (sgRNA-DFR#4, expression driven by the U6 promoter). The sequences of all the sgRNA are shown in S1 Table.

The cassette which provided the donor DNA repair template was designed with left and right homologous arms, each corresponding to the 400 bp and 392 bp sequences flanking both sides of the 1013 bp *DFR* deletion, and the *DFR* previously deleted sequence associated with a NOS terminator (nopaline synthase) followed by the *NptII* gene under the control of the NOS promoter. The DNA donor template was flanked by the target sequences of the sgRNAs DFR#3 and DFR#4. The target sequence of DFR#4 was modified in the DNA donor template to avoid unwanted DSB. The sequence of the donor DNA repair template is shown in S1 Fig.

The sgRNA cassettes with U3-sgRNA-DFR#1, U6-sgRNA-DFR#2, U3-sgRNA-DFR#3, U6-sgRNA-DFR#4 were synthesized by IDT (https://www.idtdna.com) flanked by the attB1 and attB2 Gateway sequences and introduced into pDONR207 by BP Gateway recombination. The backbones are shown in S2 Fig. The U6-sgRNA-DFR#2 cassette was cloned as a *Xho*I-*Pst*I fragment into pDONR207-U3-sgRNA-DFR#1 previously digested with *Sal*I and *Pst*I to give pDONR207-DFR#1-DFR#2. The U3-sgRNA-DFR#1-U6-sgRNA-DFR#2 cassette was then introduced into the binary vector pDe-Cas9-*NptII* by LR Gateway recombination to generate the pDe-Cas9-*NptII*-DFR#1-DFR#2 plasmid. U3-sgRNA-DFR#3 and U6-sgRNA-DFR#4 were first cloned together in the same pDONR207 vector following the same strategy previously described for pDONR207-DFR#1-DFR#2. The U3-sgRNA-DFR#3-U6-sgRNA-DFR#4 cassette and the donor template cassettes were simultaneously introduced into the binary vector pDe-Cas9-*Hpt*-GT by LR Gateway recombination to produce the pDe-Cas9-*Hpt*-GT-DFR#3-DFR#4-DFRtemp binary vector (S3 Fig).

### *Agrobacterium tumefaciens*-mediated transformation and regeneration experiments

The tomato (*Solanum lycopersicum*) WVA106 genotype was used for *Agrobacterium tumefaciens*-mediated transformation. The plants were grown in sterile conditions in a culture chamber with controlled temperatures of 22°C/18°C and a photoperiod of 16h/8h (day/night). *Agrobacterium tumefaciens*-mediated transformation of the WVA106 cultivar and T2-DFR64a lines (selected to be homozygous for the 1013 bp *DFR* deletion and without T-DNA insert) were performed as described in Mazier *et al*. (2011) [46] using cotyledon and leaf segments from 8–12 day-old seedlings and *Agrobacterium tumefaciens* strain C58 pGV2260 containing the binary vectors: pDe-Cas9-*NptII*-DFR#1-DFR#2 or pDe-Cas9-*Hpt*-GT-DFR#3-DFR#4-DFRtemp, respectively. Individual buds regenerating on 100 mg/L kanamycin-containing media were separated in culture tubes before molecular analysis. To obtain *in vitro* regenerated buds without transformation, cotyledon fragments were first placed on MS medium [47] containing 0.9 mg/L thiamine, 0.2 mg/L 2-4D, 0.1 mg/L kinetin in dark. After three days, they were transferred to regeneration media containing 2 mg/L zeatin until the buds had grown enough for phenotypic observation of *DFR* deleted plants.

### DNA extraction, PCR genotyping and sequencing of regenerating plantlets

After the *Agrobacterium tumefaciens*-mediated transformation, leaf samples from kanamycin-resistant regenerated plantlets were collected. Genomic DNA was extracted from fresh leaf samples according to Fulton and Tanksley [48]. The quality of the DNA was verified by Nanodrop and calibrated to a concentration of 500 ng/μl. To screen for the *DFR* gene deletion, T0 plantlets were genotyped using PCR with the primers DD1F and DD1R (S2 Table) designed to amplify a differentially sized fragment depending on whether the deletion is present or not (respectively 1347 bp and 2360 bp). PCR products corresponding to the deletion in the *DFR* gene were sequenced. Alignments were performed using Bioedit software [49].

The presence of the *CAS9*, *NptII*, and *Hpt* genes was verified by PCR using Cas9F and Cas9R primers (1438 bp fragment if positive) designed to amplify a region between the 3’ end of the ubi4-2 promoter and the 3’ end of the *CAS9* gene, NptIIF and NptIIR primers (593 bp fragment if positive) and HptF and HptR primers (573 bp fragment if positive), respectively (primers are listed in S2 Table).

For detection of the HDR-mediated targeted insertion, T0 plantlets were screened by PCR with the primers GT1F and GT1R (1211 bp fragment if positive), designed to detect the 5’ end of the targeted insertion, and with primers GT2F and GT2R (1338 bp fragment if positive) designed to detect the 3’ end of the targeted insertion. All PCR products were resolved on 1.5% agarose gels at 120 V for 30 min.

In an attempt to amplify the whole reconstructed *DFR* gene associated with the insertion of the *NptII* gene by HDR, a long-range PCR strategy was performed with the PrimeStar GXL DNA polymerase from Clonetech. The primers, GT3F and GT3R, were designed to amplify differentially-sized fragments depending on whether the targeted insertion is present or not (3608 bp and 2180 bp, respectively) or if there is a deletion in the *DFR* gene (1167 bp) (S2 Table)

### Anthocyanin quantification by liquid chromatography

For extraction, 2 grams of hypocotyls from 45 day-old seedlings and stems from 30 day-old *in vitro* regenerating plantlets were frozen in liquid nitrogen, ground and then lyophilized. Extraction solvent consisting of Ethanol/Water (70/30) was added to the dried samples and the extraction was performed by homogenization with an ultra-turrax and shaking at 4°C. After centrifugation, the supernatant was collected and dried in a vacuum centrifuge. 1 ml of methanol was added to the dried sample and transferred to the chromatograph. The analysis was performed with a High-Performance Liquid Chromatography system, SHIMADZU Prominence, equipped with a reversed phase C18 column (MERCK Superspher RP18 end-capped) coupled with a photodiode array detector. The anthocyanins were characterized according to their spectra and compared to known standards. For quantification, a calibration curve was obtained by injection of known concentrations of cyanidin-3-glucoside.

### T1 and T2 transgenic lines and greenhouse conditions

*In vitro* regenerated T0 plantlets were transferred to greenhouses after two weeks on rooting medium (MSO medium in which MS salts were reduced to ½). T1 and T2 transgenic lines were obtained by self-pollination of the T0 and T1 plantlets, respectively. Plants were grown in a sterilized peat soil mixture under natural light conditions with a daytime temperature of 22°C and a night time temperature of 18°C for the phenotypic observations.

## Results

### Targeted deletion at the *DFR* locus

**Fig 1:**
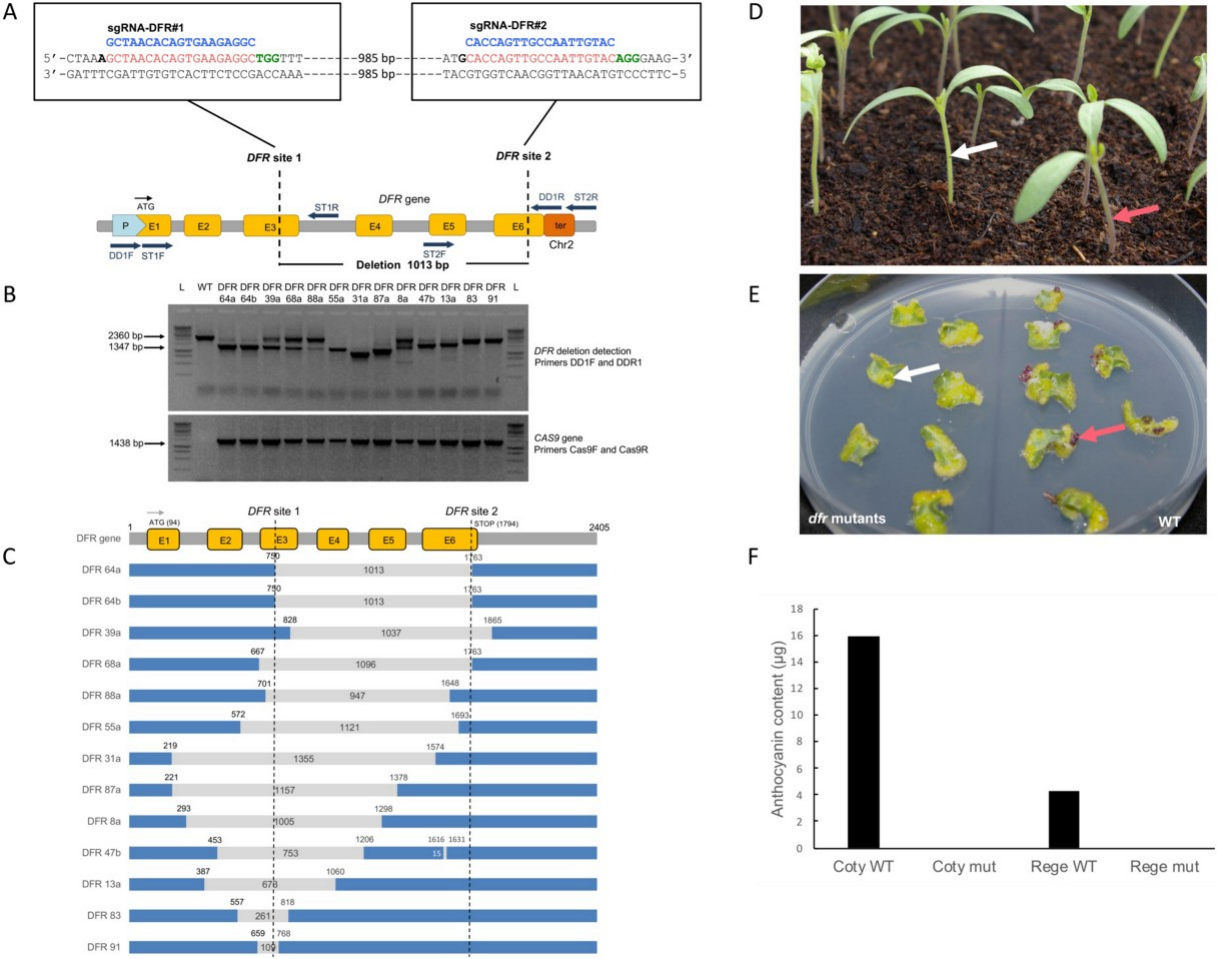
CRISPR-Cas9 mediated deletion in *DFR* marker gene. (A) Schematic illustration of the double sgRNA strategy targeting the *DFR* gene in exon 3 and exon 6 (E3 and E6 yellow boxes). The PAM site is in green, the target sequence in red and the sgRNA in blue. The promoter and terminator sequences are represented with pale blue and orange boxes, respectively. The expected length of the deletion generated by the use of these two sgRNAs is 1013 bp. The positions of the primers used for detection and sequencing are shown with dark blue arrows. (B) PCR analysis of the *DFR* locus using primers DD1F and DD1R in 13 independent T0 plants. The amplicon sizes for the wild-type genomic locus and for the predicted deletion are 2360 bp and 1347 bp, respectively (see arrows). The presence of the *CAS9* gene in the T0 plants was confirmed by PCR analysis using primers Cas9F and Cas9R. (C) Schematic representation of the deletion pattern observed in the *DFR* gene for the 13 T0 plants analysed. The numbers inside the grey section represent the deletion size and the numbers next to the blue sections represent the position of the cut in the *DFR* gene. (D) Emerging hypocotyls observed on 10 day old progeny of a T0 tomato plant that was heterozygous for the *DFR* gene deletion. Segregating T1 plantlets homozygous for the mutated *DFR* gene showed an absence of anthocyanin pigmentation in their hypocotyls (white arrow). Segregating T1 plantlets homozygous or heterozygous for the wild-type *DFR* gene showed anthocyanin pigmentation (red arrow). (E) Regeneration from cotyledon pieces two weeks after induction. Left: cotyledons of T2 plantlets issued from the *dfr* mutant T0 plant DFR64a; Right: cotyledons of wild-type WVA106. Anthocyanin pigmentation (red arrow) was observed in the first days of regeneration while it was not visible (white arrow) in *dfr* regenerating buds. (F) Anthocyanin content analysis (μg equivalent cyanidin-3-glucoside per g of fresh weight) by high-performance liquid chromatography on cotyledons and regenerating plantlets in wild type and *dfr* mutants. Coty WT: WVA106 wild-type cotyledon extract; Coty mut: *dfr* mutant extract; Rege WT: wild-type regenerating plantlet extract; Rege mut: *dfr* mutant regenerating plantlet extract. Anthocyanin contents were not detected in the mutant extracts with a minimal level of detection of 0.05 μg/g.

Although available for several tomato varieties, *dfr* mutated WVA 106 lines are not available in the tomato genetic resources (Vegetables Genetic Resources Center of UR 1052—INRA. https://www6.paca.inra.fr/gafl_eng/Vegetables-GRC/Our-Collections/Tomato-Collection/The-Solanaceae-Genetic-Resources-Network). We designed two sgRNAs: sgRNA-DFR#1, targeting exon 3, and sgRNA-DFR#2, targeting exon 6, to obtain a deletion of 1013 bp at the 3’ end of the *DFR* gene (Fig 1A). The tomato WVA106 genotype was transformed *via Agrobacterium tumefaciens* carrying the pDe-Cas9-*NptII*-DFR#1-DFR#2 binary vector. Deletions in the *DFR* gene in kanamycin resistant regenerating plantlets were identified by PCR analysis (Fig 1B). In parallel, PCR with primers specific for the *CAS9* sequence was also performed on all kanamycin resistant regenerating plantlets. Out of 91 kanamycin resistant plantlets analysed, 52 carried the *CAS9* gene and the *DFR* gene was deleted in 13 (Fig 1B). Therefore, the *DFR* gene was successfully deleted in 25% of the plantlets carrying the *CAS9* gene. The sizes of amplicons from some events were different from the wild type locus or the predicted deletion (T0 plants DFR47b, DFR13a and DFR31a for example). The amplification products corresponding to potential deletions in the *DFR* gene were sequenced and the results are shown in Fig 1C. Different deletion patterns were observed. The length of the deletions varied from 109 bp to 1355 bp. Target site 1 is missing in all deleted lines except for the DFR39a plant. Target site 2 was intact in nine plantlets out of the 13 analysed implying that the cutting efficiency was higher at the *DFR* targeted site 1. Two plantlets, DFR64a and DFR64b, contained the expected deletion of 1013 bp with the predicted Cas9 nuclease DNA cut three nucleotides upstream of the PAM motifs [50]. According to the PCR analysis several plants appeared to be homozygous for a deletion in the *DFR* gene with a unique PCR fragment (DFR55a, DFR31a, DFR87a, DFR83 and DFR91). In other plants, two PCR fragments (DFR64a, DFR64b, DFR39a, DFR68a, DFR88a, DFR47b and DFR13a) were amplified corresponding to the deleted and wild-type versions of the *DFR* locus. These plants are potentially heterozygote *dfr* mutants or chimeras consisting of mutant (heterozygotes or homozygotes) and wild-type cells. Plant DFR8a was clearly a chimera as three PCR fragments amplified for the *DFR* locus. Thus, 3.8% of the lines containing the *CAS9* gene showed the predicted deletion. After transfer to the greenhouse, independent leaf samples were collected and DNA extraction and PCR analysis were repeated to confirm the presence of the deletion.

The 13 plantlets showing a deletion in the *DFR* gene were self-pollinated. Two T0 plants, DFR55a and DFR31a, were sterile, thus T1 progeny of a total of 11 self-pollinated T0 lines were observed. The phenotype of the seedlings was recorded 10 days after germination. The T1 progeny from two T0 plants (DFR64a and DFR64b) showed a 1/4:3/4 segregation ratio for green versus purple seedlings (based on Chi2 test analysis, see S3 Table) characteristic of a deletion of the *DFR* gene present at the heterozygous stage in the T0 plants. For seven T0 progeny (DFR68a, DFR88a, DFR87a, DFR47b, DFR13a, DFR83 and DFR91) 100% of the plantlets were green. In comparison to the PCR analysis, this suggests that both alleles of the *DFR* gene were inactive, due to deletion at the homozygous stage (DFR87a, DFR83, DFR91) or bi-allelic modifications (DFR68a, DFR88a, DFR47b, DFR13a). Surprisingly, two T0 plants (DFR39a and DFR8a) produced 100% wild-type purple phenotype T1 plantlets. Molecular analysis on some of these plants failed to show any deletions in the *DFR* gene even though the deletion was detected in the T0 plants. In these cases, the deletions were probably not transmitted to the progeny due to the chimeric status of the parent.

### Selection and phenotype of the homozygous *dfr* mutant line T2-DFR64a

PCR analysis of T1 progeny of the DFR64a T0 plant (containing the expected 1013 bp deletion), displaying the green phenotype characteristic of the homozygous *dfr* mutation, was used to identify plantlets where the T-DNA had segregated away. Selfing of these individuals produced T2 seeds that were homozygous for the deletion but without T-DNA and *CAS9*. One hundred percent of these T2 seedlings displayed the green phenotype associated with the *dfr* mutation. To evaluate the suitability of this “anthocyanin-free” phenotype for landing pad purposes and its potential usefulness at early stages of the transformation experiments, a regeneration experiment was carried out to compare these mutant plantlets with a wild-type control throughout *in vitro* culture (Fig 1D and 1E). Differences between the *dfr* mutants and the wild-type, based on the colour of the regenerating structures, particularly stems and leafstalks, were visible as early as one month after starting the regeneration experiment. In addition, the *dfr* mutation appeared to have no effect on the potential of the explants to regenerate, or on the growth or fertility of the plants.

Anthocyanin levels in the wild type and *dfr* mutant were quantified using high-performance liquid chromatography on hypocotyls from 45 day-old seedlings and the stems of 30 day-old regenerating events (Fig 1F). The anthocyanins were characterized according to their spectra and compared to known standards. Four compounds with spectra corresponding to anthocyanins were detected in the control extract at 540 nm. However, in the mutant extracts, these four compounds were not found. Total anthocyanins were quantified by addition of the areas of the four peaks. The value was calculated as an equivalent of cyanidin-3-glucoside according to the calibration curve. Anthocyanin content in μg equivalent cyanidin-3-glucoside per g of fresh weight was determined in the wild type hypocotyls and *in vitro* regenerating stems, with respectively 15.9 μg/g and 4.3 μg/g of fresh weight. Anthocyanin was not detected in the mutant extracts with a minimal level of detection of 0.05 μg/g.

### Targeted gene insertion at the *DFR* landing pad

**Fig 2:**
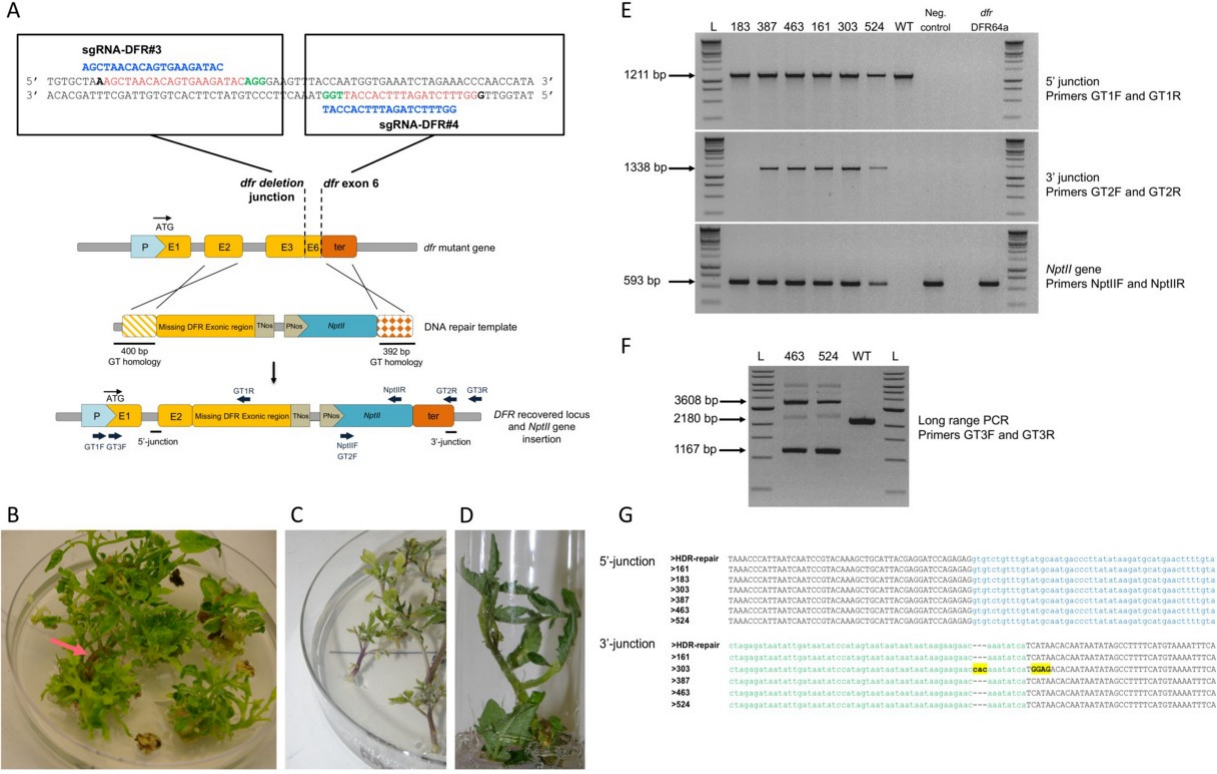
Targeted gene insertion at the *dfr* locus. (A) Schematic representation of HDR-mediated gene insertion at the *dfr* mutant locus using two sgRNAs: sgRNA-DFR#3 and sgRNA-DFR#4 targeting the *dfr* deletion junction and exon 6 (E6 yellow box). The PAM site is in green, the target sequence in red and the sgRNA in blue. The promoter and terminator sequences are represented with pale blue and orange boxes respectively (P and Ter). Gene targeting (GT) homologous sequences are shown with boxes with a hatched pattern. The *NptII* gene insertion is represented by a blue box. The primers used for detection and sequencing are shown as dark blue arrows. (B) Regenerating explants on kanamycin selective media after *Agrobacterium* transformation of the T2 plant DFR64a with a single binary vector carrying two sgRNAs and a DNA repair template containing the *DFR* sequence and *NptII* gene. The purple coloured events can be visually identified (red arrow) (C) Purple coloured plantlets regenerating on kanamycin-containing media (D) *In vitro* regenerated T0 plants with HDR-mediated-gene targeting in the *DFR* landing pad isolated in a tube. (E) PCR analysis for the detection of the precise HR with the repair template at the 5’ junction, 3’ junction and the presence of the *NptII* gene with primers GT1F and GT1R in six T0 plantlets which showed anthocyanin pigmentation. WT: wild-type; Neg. control: negative control green plantlet; *dfr* DFR64a: deleted *dfr* mutant used for the HDR-mediated experiment; 183, 387, 463, 161, 303 and 524: *in vitro* samples from T0 plantlets issued from targeted insertion events visually screened for their anthocyanin pigmentation. (F) Long range PCR with primers GT3F and GT3R on two different targeted insertions. WT: wild-type; 463 and 524: samples from T0 plantlets from event number 463 and number 524 (with anthocyanin pigmentation). (G) Sequencing of the 5’ and 3’ junctions at the *DFR* locus in T0 plantlets with recovered anthocyanin pigmentation for events 161, 183, 303, 387, 463 and 524. The sequence of the 3’ junction was not obtained for event number 183. Coloured lower-case letters indicate the end and the beginning of the homologous sequence used in the DNA donor template. Black upper-case letters show the genomic sequences surrounding the insertion. Mutations in the sequences compared to expected sequence are shown with yellow boxes and bold letters.

In order to easily integrate the DNA repair template in the same single binary vector carrying the *CAS*9 gene and the sgRNAs, we modified the pDe-Cas9 vector by inserting additional attR3 and attR4 Gateway sequences. This vector, named pDe-Cas9-*Hpt*-GT, allows for the simultaneous insertion of the DNA repair template (flanked by attL3 and attL4 Gateway sequences), and the sgRNAs (flanked by attL1 and attL2 Gateway sequences) during the LR reaction (S3 Fig). In order to induce targeted insertion at the *DFR* landing pad locus, associated with a restoration of the *DFR* gene, a donor DNA repair template was designed containing the missing *DFR* gene sequence (see M&M). To promote the insertion of the DNA repair template by homologous recombination, we included 400 bp and 392 bp long homology sequences identical to the 400 bp and 392 bp sequences flanking the deletion in the *dfr* mutant lines (Fig 2A). As proof of concept and to select regenerating buds in which the DNA repair template is integrated, we used the *NptII* gene as the inserted transgene. *NptII* was placed under the control of the NOS promoter and the 392 bp homology 3’ arm sequence was placed immediately after its stop codon. Because this 3’ homology sequence corresponds to the sequence present immediately after the *DFR* stop codon in the tomato genome, precise insertion of the *NptII* gene would potentially lead to it being under the control of the *DFR* terminator sequence. To restore *DFR* gene function, the endogenous *DFR* terminator was therefore replaced with a NOS terminator downstream of the *DFR* stop codon in the DNA donor template. In order to increase the frequency of gene targeting by the release of the donor DNA template *in planta* as previously proposed in *Arabidopsis* [24], the template was flanked by the target sequences of the sgRNAs DFR#3 and DFR#4.

The T2 DFR64a line was transformed with pDe-Cas9-*Hpt*-GT-DFR#3-DFR#4-DFRtemp, containing the *DFR* DNA repair template as previously described as well as two sgRNAs targeting the deletion junction sequence at the *DFR* locus of the DFR64a line. A total of 583 cotyledon pieces were treated and placed on regeneration media containing kanamycin for selection after the co-cultivation period. The first 597 regenerating plantlets issued from at least 265 independent regeneration events (issued from different cotyledons pieces) were systematically analysed by PCR to screen for targeted insertions. In parallel with this molecular screening, the colour of the regenerating material was noted during medium changes every two weeks. After three months (one month on selective media), a few dark-coloured regeneration structures could be observed on cotyledons (Fig 2B, 2C and 2D). A total of six independent purple coloured regenerating plantlets was obtained (events 161, 183, 303, 387, 463 and 524). Targeted transgene insertion at the *DFR* locus was confirmed by PCR in these six plantlets (Fig 2E and 2F and S4 Table). PCR analysis of the other 597 green coloured plantlets confirmed that the transgene had inserted randomly in these lines. The amplification products were sequenced and confirmed the targeted insertion at the *DFR* landing pad. Five out of the six events (T0 #161, 303, 387, 463 and 524) were issued from HDR on both sides (Fig 2G) and PCR amplification of the full length *DFR* and *NptII* gene insertion block was obtained for events 463 and 524 (Fig 2F). For event number 183, no amplification product was obtained with primers GT2F and GT2R (3’ side) while PCR amplification of the 5’ border was positive, corresponding to the reconstruction of the *DFR* gene. As 463 out of the 583 agro-infected cotyledons were able to regenerate kanamycin resistant plantlets, we can estimate that the efficiency of targeted insertion was 1.29% of the transformed explants.

The T1 progeny of four T0 plants with a targeted transgene insertion (# 183, 303, 387 and 463) were harvested, sown and the colour of their hypocotyls observed. For two events, T0 #303 and 183, hypocotyls of the T1 progeny (n = 50) were green, suggesting that the transgene was not transmitted from the T0 plant to the progeny. PCR analysis confirmed that the transgene was not present in these T1 plants. Non-transmission of the targeted transgene from the T0 plants to their T1 progenies could be due to the chimeric status of the transgene targeted insertion in the T0 plants #303 and #183. Hypocotyl colour and PCR analysis (not shown) in the T1 progenies (n = 81 and 66) of T0 plants #387 and 463 respectively, showed that the targeted transgene was transmitted as a Mendelian trait in these plants.

## Discussion

The CRISPR-Cas9 system has been demonstrated to be an efficient and versatile tool in many organisms, including plants, for gene knock-out through NHEJ-mediated repair. In the present study, we confirmed previous reports on the efficiency of the CRISPR-Cas9 system to generate deletions in the tomato genome [3,21]. In our case, we obtained a 1013 bp deletion at the *DFR* locus with an efficiency of 25% by using a double sgRNA strategy. Two T0 plants showed exactly the expected and desired deletion in the *DFR* gene, and 11 other T0 transformants carried deletions ranging from 109 bp to 1355 bp. From these, 53.8% (7 out of 13) were bi-allelic or homozygous mutants.

We have shown here that deletion in the *DFR* gene at the homozygous stage led to a complete disruption of DFR function and to a seedling with green hypocotyls that could be easily visually discriminated from the purple coloured hypocotyl of the wild-type. These *dfr* homozygous line were the first step in our strategy aiming at using the *DFR* locus as a landing pad. We showed here that such mutant lines are easy to visually screen and obtain using a simple CRISPR-Cas9 strategy.

Next, using the *dfr* mutant line as starting material, we isolated six independent targeted insertions at the *DFR* landing pad. The rare HDR mediated events obtained after transformation were easy to identify due to the recovery of DFR function and the associated purple colour of the regenerated plantlets. The deleted *DFR* gene was repaired using a DNA template carrying the missing *DFR* sequences and 400 bp homology arms. Sequencing and molecular analysis of the purple-coloured T0 plantlets confirmed the precise HDR-mediated insertion of the DNA donor template and the recovery of the functional *DFR* gene.

In 1.29% of the transformed plants (6 out of 463) HDR-mediated insertion of the transgene occurred. This number is close to the frequency of HDR-mediated gene replacement obtained in *Arabidopsis* for example (frequency up to 0.8%) [51]. This low efficiency of targeted integration through *Agrobacterium*-mediated transformation was previously reported [24,27,41] and could be related to the low number of T-DNA copies delivered into the plant cells. Strategies based on particle bombardment or DNA virus-based replicons were reported to be valuable for HDR-mediated gene replacement in many plant species such as rice, maize, soybean, wheat, cotton and tomato, for reviews see [28,52]. Nevertheless, those direct DNA transfer techniques or genetically modified viruses are not necessarily accessible in all laboratories and delivery of DNA into the cell *via Agrobacterium* mediated transformation is still the method of choice for a great number of plants, including tomato. Different strategies have been proposed to increase the low frequency of HDR-mediated gene targeting through *Agrobacterium* transformation, such as inhibiting the NHEJ pathway by knocking-out the *lig4* gene or using a positive–negative selection (PNS) system, for review see [31]. Unfortunately, the knock-out of genes involved in the NHEJ repair pathway can also have an impact on transformation stability and the capacity of the plant to repair DNA damage, leading to unwanted mutations. The PNS system involves the use of antibiotic resistance markers which are generally not desirable in crop breeding.

Other strategies based on the use of modified Cas9 (paired-nickase) or of the CPF1 nuclease have also been proposed to increase gene targeting frequency (8% to 14.5%) [24,41,53]. Finally, a strategy based on the release, *via* CRISPR-Cas9, of the donor template from the T-DNA in order to facilitate the accessibility of the donor template to the targeted locus has been described in *Arabidopsis* [24]. Here, we used this approach to increase the frequency of targeted insertions in tomato. We demonstrated that the combination of this strategy with the set-up of a visual screen, to easily detect the HDR-mediated gene insertion events, makes transgene targeted insertion feasible for crop breeding.

In our system, strict correlation was found between the restoration of a functional *DFR* gene, as revealed by a change in the colour of the plantlets, and the HDR-mediated gene targeting of the transgene at this locus. Thus, in addition to being an efficient landing pad for targeted transgene insertion, the *DFR* locus also provides an alternative endogenous reporter gene to detect HDR-mediated gene targeting and estimate its efficiency.

In conclusion, the strategy presented here has multiple advantages. The transgene targeting insertion is based on a simple and routine *A*. *tumefaciens* transformation experiment involving a single binary vector. Plants with a transgene targeted integration are easily selected by visual screening of regenerating seedlings. The *DFR* gene can be used as an HDR-based visual marker for gene knock-in improvements in which only a few bases must be changed. The DFR function is very well conserved in many plants, thus the proposed strategy is most likely applicable to other crop species.

## Supporting information

S1 Fig *DFR* donor repair template used for HDR-mediated gene reconstruction. AttL3 and attL4 are colorized with purple, target sequence sgRNA *dfr*#3 is colorized with black and white letters, target sequence sgRNA *dfr*#4 is represented by grey color and black letters. The homology left and right arms are represented in yellow and orange. The deleted sequence is represented in dark yellow and white letters. The promoter Nos is represented in brown, and the terminator Nos is represented in pale brown and white letters. The *NptII* gene is represented in blue. The synonymous mutations designed to disrupt the sgRNA target sequence *dfr* are represented in red.
(DOCX)

S2 Fig Backbones sgRNA-U3 and sgRNA-U6 used in tomato. In green, the tracrRNA motif, in orange and blue the promoter U6 and U3. In purple, the Gateway sequences attB1 and attB2. The stars represent the target sequence of the form 5’-A-N_(19)_NGG-3’ with the respect to the U3 promoter and of the form 5’-G- N_(19)_NGG-3’ with the respect to the U6 promoter. Underlined, restriction enzyme sites used for the cloning of the double sgRNA (*Xho*I -*Sal*I). The *Pst*I site is present in the pDONR207 sequence.
(DOCX)

S3 Fig Schematic representation of the constructions used for targeted insertion at *DFR* landing pad. The U3 and U6 promoter are represented by blue and red boxes. The *CAS9* gene is represented by a pink box. The left border (LB) and right border (RB) are represented with yellow boxes. The *NptII* gene is represented with a blue box, while the *Hpt* gene is represented by a brown box.
(DOCX)

S1 Table List of the sgRNA used. (DOCX)

S2 Table List of the primers used. (DOCX)

S3 Table Summary of all events obtained by self-pollination and phenotypic observations. (DOCX)

S4 Table Summary of HDR-mediated gene recovering and gene insertion at the *DFR* locus. (DOCX)

## Abbreviations

Alt-EJ: alternative non-homologous end-joining
Cas9: CRISPR-associated protein
C-NHEJ: classical non- homologous end-joining
CRISPR: Clustered Regularly Interspaced Short Palindromic Repeats
DFR: dihydroflavonol 4-reductase
DSB: double-strand DNA breaks
HDR: homology-directed repair
HR: homologous recombination
GOI: gene of interest
MMEJ: microhomology-mediated end-joining
NHEJ: non-homologous end-joining
PAM: protospacer adjacent motif
TALEN: Transcription Activator Like effector nucleases
sgRNA: single guide RNA
ZFN: zinc finger nucleases
