# Supplementary material for: The DFR locus: A smart landing pad for targeted transgene insertion in tomato
Source: PLoS One. 2018 Dec 6;13(12):e0208395. doi: 10.1371/journal.pone.0208395 (PMC6283539; doi:10.1371/journal.pone.0208395)
Supplement: S1 Fig — AttL3 and attL4 are colorized with purple, target sequence sgRNA dfr#3 is colorized with black and white letters, target sequence sgRNA dfr#4 is represented by grey color and black letters. The homology left and right arms are represented in yellow and orange. The deleted sequence is represented in dark yellow and white letters. The promoter Nos is represented in brown, and the terminator Nos is represented in pale brown and white letters. The NptII gene is represented in blue. The synonymous mutations designed to disrupt the sgRNA target sequence dfr are represented in red. (DOCX) [file pone.0208395.s001.docx]

CAACTTTATTATACAAAGTTGGCATTATAAAAAAGCATTGCTTATCAATTTGTTGCAACGAACAGGTCACTATCAGTCAAAATAAAATCATTATTTactagtCCAATGGTGAAATCTAGAAACCCAGCTAACACAGTGAAGATACAGGccatggGGTGTCTGTTTGTATGCAATGACCCTTATATAAGATGCATGAACTTTTGTAGAGAACCAGAAGAAGGTGAAACATCTGTTGGAATTGCCAAAAGCTGATACAAACTTAACGCTGTGGAAAGCTGACTTGGCAGTGGAAGGAAGCTTTGATGAAGCCATTCAAGGCTGTCAAGGAGTATTTCATGTGGCTACACCCATGGATTTCGAGTCCAAGGATCCAGAGGTACTATAATGTAACAGAGTAGCTTATCAATACTTATGGACTTGTTACATTTCTGGATGATTAATACTAAAGTAACTCTCATGACGTTGATATACGTGACAGAACGAAGTAATCAAACCAACAGTCAGGGGAATGTTAAGTATCATAGAATCATGTGCTAAAGCTAACACAGTGAAGAGGCTGGTTTTCACTTCATCTGCTGGAACTCTTGATGTCCAAGAGGACCAAAAACTCTTCTATGACGAGACCAGCTGGAGCGATTTGGACTTCATATATGCTAAGAAGATGACAGGCTGGGTTCGTTTGGCTATTCTTCTCTTTTAAGAATACCCTTTCTATGTTTAAAAAGAAAAAAAAGTATTTTCATCACATTGCTTTGTGAATTTAATTTGATTGCAGATGTATTTTGTTTCCAAGATACTGGCAGAGAAGGCTGCAATGGAAGAAGCTAGAAAGAATAACATTGATTTCATTAGCATCATACCACCACTGGTTGTTGGTCCATTCATCACATCTACGTTCCCACCAAGCTTAATCACTGCCCTTTCACTAATTACCGGTATGATTAATCACAGTCAAGAAGACAGTCAAATCCTTTAATTCCAGTTATTACTTAATTAACAGCATTTCACAATCATGGGCTTAATCAGATAGAGATGTGCAGGGAATGAAGCTCACTACGGCATCATTAAACAAGGTCAATATGTGCATTTGGATGATCTTTGTGAGGCTCATATATTCCTGTATGAGCACCCCAAGGCAGAGGGAAGATTCATTTGCTCATCCCATCATGCTATCATCTACGATGTGGCTAAGATGGTGCGACAGAAATGGCCAGAGTACTATGTTCCTACTGAGTAAGCCACTCTGCTCCTCTGTATTCCCAAGTATAATTGGCTCCTTCGTAGAGTGATGGATTGGTAACTCAATCTGGTAAAATAACAGGTTTAAGGGTATCGATAAGGACTTGCCCGTAGTGTCTTTTTCATCAAAGAAGCTTATGGATATGGGGTTTCAATTCAAACACACTTTGGAGGATATGTATAAAGGGGCCATTGAGACTTGCCGACAGAAGCAATTGCTTCCCTTTTCTACCCGAAGCACTGCAGACAATGGAAAAGACAAAGAAGCAATTCCCATTTCTACTGAAAACTATTCAAGTGGCAAGGAGAATGCACCAGTTGCCAATTGCACAGGGAAGTTTACTAATGGTGAAATCTAGGCGGGACTCTGGGGTTCGGACTCTAGCTAGAGTCAAGCAGATCGTTCAAACATTTGGCAATAAAGTTTCTTAAGATTGAATCCTGTTGCCGGTCTTGCGATGATTATCATATAATTTCTGTTGAATTACGTTAAGCATGTAATAATTAACATGTAATGCATGACGTTATTTATGAGATGGGTTTTTATGATTAGAGTCCCGCAATTATACATTTAATACGCGATAGAAAACAAAATATAGCGCGCAAACTAGGATAAATTATCGCGCGCGGTGTCATCTATGTTACTAGATCGACCGGCATGCAAGCTGGCGGCCGCTTCGAACTCGAGTCATACATGAGAATTAAGGGAGTCACGTTATGACCCCCGCCGATGACGCGGGACAAGCCGTTTTACGTTTGGAACTGACAGAACCGCAACGTTGAAGGAGCCACTCAGCCGCGGGTTTCTGGAGTTTAATGAGCTAAGCACATACGTCAGAAACCATTATTGCGCGTTCAAAAGTCGCCTAAGGTCACTATCAGCTAGCAAATATTTCTTGTCAAAAATGCTCCACTGACGTTCCATAAATTCCCCTCGGTATCCAATTAGAGTCTCATATTCACTCTCAACTCGATCGAGGCATGATTGAACAAGATGGATTGCACGCAGGTTCTCCGGCCGCTTGGGTGGAGAGGCTATTCGGCTATGACTGGGCACAACAGACAATCGGCTGCTCTGATGCCGCCGTGTTCCGGCTGTCAGCGCAGGGGCGCCCGGTTCTTTTTGTCAAGACCGACCTGTCCGGTGCCCTGAATGAACTCCAAGACGAGGCAGCGCGGCTATCGTGGCTGGCCACGACGGGCGTTCCTTGCGCAGCTGTGCTCGACGTTGTCACTGAAGCGGGAAGGGACTGGCTGCTATTGGGCGAAGTGCCGGGGCAGGATCTCCTGTCATCTCACCTTGCTCCTGCCGAGAAAGTATCCATCATGGCTGATGCAATGCGGCGGCTGCATACGCTTGATCCGGCTACCTGCCCATTCGACCACCAAGCGAAACATCGCATCGAGCGAGCACGTACTCGGATGGAAGCCGGTCTTGTCGATCAGGATGATCTGGACGAAGAGCATCAGGGGCTCGCGCCAGCCGAACTGTTCGCCAGGCTCAAGGCGCGGATGCCCGACGGCGAGGATCTCGTCGTGACCCACGGCGATGCCTGCTTGCCGAATATCATGGTGGAAAATGGCCGCTTTTCTGGATTCATCGACTGTGGCCGGCTGGGTGTGGCGGACCGCTATCAGGACATAGCGTTGGCTACCCGTGATATTGCTGAAGAGCTTGGCGGCGAATGGGCTGACCGCTTCCTCGTGCTTTACGGTATCGCCGCTCCCGATTCGCAGCGCATCGCCTTCTATCGCCTTCTTGACGAGTTCTTCTGAAAACCCAACCATACATAATAAAAAGGCGAGCTACCTCACCAACATGTTCTTGGTTCTTGATGGCTGCTTGAGGTTTTTCAACACAACATGAGTAAAATGGTCAATAAATATGGATATTCTCATCTTGCTTGATTCGGGCAGGTAGGATATCAGAGAATTCTAGGTATATTTTGTAGGGAAAAAAAACTAGAATCTCTTCAGGATCTGGATCCTAATACAAGGACATATTAATATTCCATTTTATAAACTTTGCACAAGCAAGATCTAATTTTATGGAAGAACTGAAGGCTCTCGCGTATCATTTGCCAAAATAATTACCATCTTTTGTAATCATCTTGGAAACTAGAGATAATATTGATAATATCCATAGTAATAATAATAATAAGAAGAACCACAAATATCATGGAGATCTCCAATGGTGAAATCTAGAAACCCAGCTAACACAGTGAAGATACAGGactagtAAATAATGATTTTATTTTGACTGATAGTGACCTGTTCGTTGCAACAAATTGATAAGCAATGCTTTTTTATAATGCCAACTTTGTATAGAAAAGTTG

**S1 Fig. *DFR* donor repair template used for HDR-mediated gene reconstruction.**

AttL3 and attL4 are colorized with purple, target sequence sgRNA *dfr*#3 is colorized with black and white letters, target sequence sgRNA *dfr*#4 is represented by grey color and black letters. The homology left and right arms are represented in yellow and orange. The deleted sequence is represented in dark yellow and white letters. The promoter Nos is represented in brown, and the terminator Nos is represented in pale brown and white letters. The *NptII* gene is represented in blue. The synonymous mutations designed to disrupt the sgRNA target sequence *dfr* are represented in red.

sgRNA-U6 backbone

ggggacaagtttgtacaaaaaagcaggcttcGAGCTCCTCGAGACATGTATTAACGGTGATCAATTGGTTAAAAAAAAGTTTATTATTAAAATGATAAATCTTTTTAATTTATAGTATATTTATGTAAGTTTTCACGTTGAGTAAATAGCGAAGAAGTTGGGCCCAACCAAGTAAAATAAGAAGGCCGGGCCATTACAATTAAGTCGTCACACAACTGGGCTTCATTGAAAAAAGCGCAAAACCGATTCCAGGCCCGTGTTAGCATGAAGACTCAACTCAACCAGAGATTTCTCCCTCATCGCTTACAGAAAAAAGCTATATGCTGTTTATATTGCGAATCTAACAGTGTAGTTTg*******************GTTTTAGAGCTAGAAATAGCAAGTTAAAATAAGGCTAGTCCGTTATCAACTTGAAAAAGTGGCACCGAGTCGGTGCTTTTTTTGAGCTCGAATTCgacccagcttTcttgtacaaagtggtcccc

sgRNA-U3 backbone

ggggacaagtttgtacaaaaaagcaggcttcGAGCTCGAATTCTGAAACTTTACAAGTGAATTATTATGGAGTTCATGGCAACTGCTATGGAGTTTTTCCTACTGGGAATTGGAACGGTTTCTACGAAATTAACTGTCCACACGTTAAAAATATAAATTAATGCGTAATTGTTATTTTTTCTATAACAAATAAAAAACTGAAATACGACATAAATTTTATTACTTTAATTGCACTTTAGCCTTAGAGATATTGCGTTGTAGTCGGCGTAGGTGTGTCAGGGGCCAATATATTGTTCCCACATCGGCAGTGCAGCACATAAACTCTAGCGTTATAAGAATCTATCCACTATCAACGGTCa*******************GTTTTAGAGCTAGAAATAGCAAGTTAAAATAAGGCTAGTCCGTTATCAACTTGAAAAAGTGGCACCGAGTCGGTGCTTTTTTTGAGCTCGTCgacccagctttcttgtacaaagtggtcccc

**S2 Fig. Backbones sgRNA-U3 and sgRNA-U6 used in tomato.**

In green, the tracrRNA motif, in orange and blue the promoter U6 and U3. In purple, the Gateway ® sequences attB1 and attB2. The stars represent the target sequence of the form 5’-A-N_(19)_NGG-3’ with the respect to the U3 promoter and of the form 5’-G- N_(19)_NGG-3’ with the respect to the U6 promoter. Underlined, restriction enzyme sites used for the cloning of the double sgRNA (*Xho*I -*Sal*I). The *Pst*I site is present in the pDONR207 sequence.

**S1 Table. List of the sgRNA used.**

| **Target** | **Role** | **gRNA ID** | **Sequence (5’-3’)** |
| --- | --- | --- | --- |
| *DFR* exon 3 | deletion | sgRNA DFR#1 | GCTAACACAGTGAAGAGGC |
| *DFR* exon 6 | deletion | sgRNA DFR#2 | CACCAGTTGCCAATTGTAC |
| *dfr* deleted junction and Donor template | DSB insertion  Release of DNA donor template | sgRNA DFR#3 | AGCTAACACAGTGAAGATAC |
| *dfr* exon 6 and donor template | DSB insertion  Release of DNA donor template | sgRNA DFR#4 | GCTAACACAGTGAAGATAC |

**S2 Table. List of the primers used.**

| **Target** | **Primer ID** | | **Sequence (5’-3’)** | | | **Size (bp)** |  |
| --- | --- | --- | --- | --- | --- | --- | --- |
| Detection *DFR* deletion | DD1F | | CACTTAAGGTTAAATTTGCTGACTC | | | 2360 if deletion 1347 |  |
|  | DD1R | | TGGAACAGATTCAACTCTGAGG | | |  |  |
| Sequencing targeted *DFR* site 1 | ST1F | | ACTCTCCTCCGAAGACGACA | | | 892 |  |
|  | ST1R | | TTCTTCCATTGCAGCCTTCT | | |  |  |
| Sequencing targeted *DFR* site 2 | ST2F | | TGTGCATTTGGATGATCTTTG | | | 794 |  |
|  | ST2R | | CCATAAAATTAGATCTTGCTTGTGC | | |  |  |
| *CAS9* gene detection | CAS9F | | TCCCTTACTACGTGGGACCTC | | | 1438 |  |
|  | CAS9R | | ATCTGCCTGGTTTCCACAAG | | |  |  |
| *NptII* detection | NptIIF | | AGACAATCGGCTGCTCTGAT | | | 593 |  |
|  | NptIIR | | AGCCAACGCTATGTCCTGAT | | |  |  |
| *HptII* detection | HptF | | ataggtcaggctctcgctga | | | 573 |  |
|  | HptR | | ATCATACATGAGAATTAAGGG | | |  |  |
| 5’ repair template insertion | GT1F | | ATCGTTGTAATTGTCATATACTAGTGG | | | 1211 |  |
|  | GT1R | | CCCATGATTGTGAAATGCTG | | |  |  |
| 3’ repair template insertion | GT2F | | AGACAATCGGCTGCTCTGAT | | | 1338 |  |
|  | GT2R | | TGGAACAGATTCAACTCTGAGG | | |  |  |
| From either side of the donor template | GT3F | | CTGAAAATGGCAAGTGAAGCTCA | | | 3608 if insertion  2180  1167 if deletion |  |
|  | GT3R | | AGCCATGTTAGCCCATTTTCTTT | | |  |  |
|  | |  | |  |  | | |
|  | |  | |  |  | | |

**S3 Table. Summary of all events obtained by self-pollination and phenotypic observations.**

| **Mutant Lines** | **Modification** | **Number of plants T1** | **Green plantlets** | **Red plantlets** | **X²obs** | **Comments** |
| --- | --- | --- | --- | --- | --- | --- |
| **DFR64a** | Deletion of 1013 bp | 72 | 18 | 54 | 0,000 |  |
| **DFR64b** | Deletion of 1013 bp | 69 | 23 | 46 | 2,556 |  |
| **DFR39a** | Deletion of 989 bp | 100 | 0 | **100** |  |  |
| **DFR68a** | Deletion of 1096 bp | 100 | **100** | 0 |  |  |
| **DFR88a** | Deletion of 949 bp | 100 | **100** | 0 |  |  |
| **DFR55a** | Deletion of 1121 bp |  |  |  |  | **No seed** |
| **DFR31a** | Deletion of 1355 bp |  |  |  |  | **No seed** |
| **DFR87a** | Deletion of 1157 bp | 100 | **100** | 0 |  |  |
| **DFR8a** | Deletion of 1015 bp | 100 | 0 | **100** |  |  |
| **DFR47b** | Deletion of 753 bp | 100 | **100** | 0 |  |  |
| **DFR13a** | Deletion of 673 bp | 100 | **100** | 0 |  |  |
| **DFR83** | Deletion of 261 bp | 100 | **100** | 0 |  |  |
| **DFR91** | Deletion of 109 bp | 100 | **100** | 0 |  |  |

**S4 Table. Summary of HDR-mediated gene recovering and gene insertion at the *DFR* locus.**

|  | Independant regeneration events | Plantlets analyzed | Explant regenerated on selective media | Events with a red color  No (%) | Two junction PCR-positive events  No (%) |
| --- | --- | --- | --- | --- | --- |
| sgRNA+Cas9+ donor template, all in single vector, *agrobacterium* mediated delivery | 265 | 597 | 463 | 6 (1.29%*) | 5 (0.84%) |

* Percentage was determined in relation to the explant regenerated on selective media.
