# Supplementary material for: The DFR locus: A smart landing pad for targeted transgene insertion in tomato
Source: PLoS One. 2018 Dec 6;13(12):e0208395. doi: 10.1371/journal.pone.0208395 (PMC6283539; doi:10.1371/journal.pone.0208395)
Supplement: S2 Fig — In green, the tracrRNA motif, in orange and blue the promoter U6 and U3. In purple, the Gateway sequences attB1 and attB2. The stars represent the target sequence of the form 5’-A-N(19)NGG-3’ with the respect to the U3 promoter and of the form 5’-G- N(19)NGG-3’ with the respect to the U6 promoter. Underlined, restriction enzyme sites used for the cloning of the double sgRNA (XhoI -SalI). The PstI site is present in the pDONR207 sequence. (DOCX) [file pone.0208395.s002.docx]

sgRNA-U6 backbone

ggggacaagtttgtacaaaaaagcaggcttcGAGCTCCTCGAGACATGTATTAACGGTGATCAATTGGTTAAAAAAAAGTTTATTATTAAAATGATAAATCTTTTTAATTTATAGTATATTTATGTAAGTTTTCACGTTGAGTAAATAGCGAAGAAGTTGGGCCCAACCAAGTAAAATAAGAAGGCCGGGCCATTACAATTAAGTCGTCACACAACTGGGCTTCATTGAAAAAAGCGCAAAACCGATTCCAGGCCCGTGTTAGCATGAAGACTCAACTCAACCAGAGATTTCTCCCTCATCGCTTACAGAAAAAAGCTATATGCTGTTTATATTGCGAATCTAACAGTGTAGTTTg*******************GTTTTAGAGCTAGAAATAGCAAGTTAAAATAAGGCTAGTCCGTTATCAACTTGAAAAAGTGGCACCGAGTCGGTGCTTTTTTTGAGCTCGAATTCgacccagcttTcttgtacaaagtggtcccc

sgRNA-U3 backbone

ggggacaagtttgtacaaaaaagcaggcttcGAGCTCGAATTCTGAAACTTTACAAGTGAATTATTATGGAGTTCATGGCAACTGCTATGGAGTTTTTCCTACTGGGAATTGGAACGGTTTCTACGAAATTAACTGTCCACACGTTAAAAATATAAATTAATGCGTAATTGTTATTTTTTCTATAACAAATAAAAAACTGAAATACGACATAAATTTTATTACTTTAATTGCACTTTAGCCTTAGAGATATTGCGTTGTAGTCGGCGTAGGTGTGTCAGGGGCCAATATATTGTTCCCACATCGGCAGTGCAGCACATAAACTCTAGCGTTATAAGAATCTATCCACTATCAACGGTCa*******************GTTTTAGAGCTAGAAATAGCAAGTTAAAATAAGGCTAGTCCGTTATCAACTTGAAAAAGTGGCACCGAGTCGGTGCTTTTTTTGAGCTCGTCgacccagctttcttgtacaaagtggtcccc

**S2 Fig. Backbones sgRNA-U3 and sgRNA-U6 used in tomato.**

In green, the tracrRNA motif, in orange and blue the promoter U6 and U3. In purple, the Gateway ® sequences attB1 and attB2. The stars represent the target sequence of the form 5’-A-N_(19)_NGG-3’ with the respect to the U3 promoter and of the form 5’-G- N_(19)_NGG-3’ with the respect to the U6 promoter. Underlined, restriction enzyme sites used for the cloning of the double sgRNA (*Xho*I -*Sal*I). The *Pst*I site is present in the pDONR207 sequence.
