# Supplementary figures and images for: The DFR locus: A smart landing pad for targeted transgene insertion in tomato
Source: PLoS One. 2018 Dec 6;13(12):e0208395. doi: 10.1371/journal.pone.0208395 (PMC6283539; doi:10.1371/journal.pone.0208395)

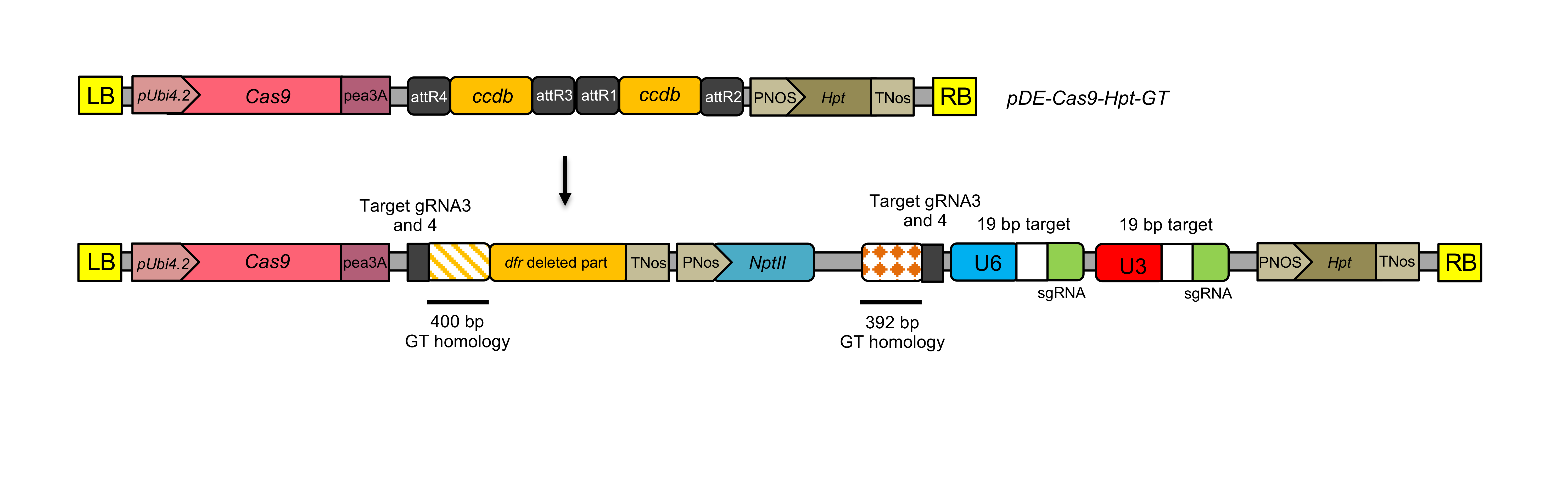

Supplement: S3 Fig — The U3 and U6 promoter are represented by blue and red boxes. The CAS9 gene is represented by a pink box. The left border (LB) and right border (RB) are represented with yellow boxes. The NptII gene is represented with a blue box, while the Hpt gene is represented by a brown box. (DOCX) [file pone.0208395.s003.docx]
