# Supplementary material for: The DFR locus: A smart landing pad for targeted transgene insertion in tomato
Source: PLoS One. 2018 Dec 6;13(12):e0208395. doi: 10.1371/journal.pone.0208395 (PMC6283539; doi:10.1371/journal.pone.0208395)
Supplement: S1 Table — (DOCX) [file pone.0208395.s004.docx]

**S1 Table. List of the sgRNA used.**

| **Target** | **Role** | **gRNA ID** | **Sequence (5’-3’)** |
| --- | --- | --- | --- |
| *DFR* exon 3 | deletion | sgRNA DFR#1 | GCTAACACAGTGAAGAGGC |
| *DFR* exon 6 | deletion | sgRNA DFR#2 | CACCAGTTGCCAATTGTAC |
| *dfr* deleted junction and Donor template | DSB insertion  Release of DNA donor template | sgRNA DFR#3 | AGCTAACACAGTGAAGATAC |
| *dfr* exon 6 and donor template | DSB insertion  Release of DNA donor template | sgRNA DFR#4 | GCTAACACAGTGAAGATAC |
