# Supplementary material for: The DFR locus: A smart landing pad for targeted transgene insertion in tomato
Source: PLoS One. 2018 Dec 6;13(12):e0208395. doi: 10.1371/journal.pone.0208395 (PMC6283539; doi:10.1371/journal.pone.0208395)
Supplement: S2 Table — (DOCX) [file pone.0208395.s005.docx]

**S2 Table. List of the primers used.**

| **Target** | **Primer ID** | | **Sequence (5’-3’)** | | | **Size (bp)** |  |
| --- | --- | --- | --- | --- | --- | --- | --- |
| Detection *DFR* deletion | DD1F | | CACTTAAGGTTAAATTTGCTGACTC | | | 2360 if deletion 1347 |  |
|  | DD1R | | TGGAACAGATTCAACTCTGAGG | | |  |  |
| Sequencing targeted *DFR* site 1 | ST1F | | ACTCTCCTCCGAAGACGACA | | | 892 |  |
|  | ST1R | | TTCTTCCATTGCAGCCTTCT | | |  |  |
| Sequencing targeted *DFR* site 2 | ST2F | | TGTGCATTTGGATGATCTTTG | | | 794 |  |
|  | ST2R | | CCATAAAATTAGATCTTGCTTGTGC | | |  |  |
| *CAS9* gene detection | CAS9F | | TCCCTTACTACGTGGGACCTC | | | 1438 |  |
|  | CAS9R | | ATCTGCCTGGTTTCCACAAG | | |  |  |
| *NptII* detection | NptIIF | | AGACAATCGGCTGCTCTGAT | | | 593 |  |
|  | NptIIR | | AGCCAACGCTATGTCCTGAT | | |  |  |
| *HptII* detection | HptF | | ataggtcaggctctcgctga | | | 573 |  |
|  | HptR | | ATCATACATGAGAATTAAGGG | | |  |  |
| 5’ repair template insertion | GT1F | | ATCGTTGTAATTGTCATATACTAGTGG | | | 1211 |  |
|  | GT1R | | CCCATGATTGTGAAATGCTG | | |  |  |
| 3’ repair template insertion | GT2F | | AGACAATCGGCTGCTCTGAT | | | 1338 |  |
|  | GT2R | | TGGAACAGATTCAACTCTGAGG | | |  |  |
| From either side of the donor template | GT3F | | CTGAAAATGGCAAGTGAAGCTCA | | | 3608 if insertion  2180  1167 if deletion |  |
|  | GT3R | | AGCCATGTTAGCCCATTTTCTTT | | |  |  |
|  | |  | |  |  | | |
|  | |  | |  |  | | |
