# Supplementary material for: The DFR locus: A smart landing pad for targeted transgene insertion in tomato
Source: PLoS One. 2018 Dec 6;13(12):e0208395. doi: 10.1371/journal.pone.0208395 (PMC6283539; doi:10.1371/journal.pone.0208395)
Supplement: S3 Table — (DOCX) [file pone.0208395.s006.docx]

**S3 Table. Summary of all events obtained by self-pollination and phenotypic observations.**

| **Mutant Lines** | **Modification** | **Number of plants T1** | **Green plantlets** | **Red plantlets** | **X²obs** | **Comments** |
| --- | --- | --- | --- | --- | --- | --- |
| **DFR64a** | Deletion of 1013 bp | 72 | 18 | 54 | 0,000 |  |
| **DFR64b** | Deletion of 1013 bp | 69 | 23 | 46 | 2,556 |  |
| **DFR39a** | Deletion of 989 bp | 100 | 0 | **100** |  |  |
| **DFR68a** | Deletion of 1096 bp | 100 | **100** | 0 |  |  |
| **DFR88a** | Deletion of 949 bp | 100 | **100** | 0 |  |  |
| **DFR55a** | Deletion of 1121 bp |  |  |  |  | **No seed** |
| **DFR31a** | Deletion of 1355 bp |  |  |  |  | **No seed** |
| **DFR87a** | Deletion of 1157 bp | 100 | **100** | 0 |  |  |
| **DFR8a** | Deletion of 1015 bp | 100 | 0 | **100** |  |  |
| **DFR47b** | Deletion of 753 bp | 100 | **100** | 0 |  |  |
| **DFR13a** | Deletion of 673 bp | 100 | **100** | 0 |  |  |
| **DFR83** | Deletion of 261 bp | 100 | **100** | 0 |  |  |
| **DFR91** | Deletion of 109 bp | 100 | **100** | 0 |  |  |
