# Supplementary material for: The DFR locus: A smart landing pad for targeted transgene insertion in tomato
Source: PLoS One. 2018 Dec 6;13(12):e0208395. doi: 10.1371/journal.pone.0208395 (PMC6283539; doi:10.1371/journal.pone.0208395)
Supplement: S4 Table — (DOCX) [file pone.0208395.s007.docx]

**S4 Table. Summary of HDR-mediated gene recovering and gene insertion at the *DFR* locus.**

|  | Independant regeneration events | Plantlets analyzed | Explant regenerated on selective media | Events with a red color  No (%) | Two junction PCR-positive events  No (%) |
| --- | --- | --- | --- | --- | --- |
| sgRNA+Cas9+ donor template, all in single vector, *agrobacterium* mediated delivery | 265 | 597 | 463 | 6 (1.29%*) | 5 (0.84%) |

* Percentage was determined in relation to the explant regenerated on selective media.
